# Eccentric training effects on hamstring muscles in oral contraceptive users and naturally menstruating women

**DOI:** 10.1007/s00421-025-05753-x

**Published:** 2025-03-15

**Authors:** Olivier Seynnes, Antoine Nordez, Lilian Lacourpaille, Eirik Hesseberg, Ingvild Vesterhus, Ken Fjeldberg, Martin Kvalvik Engstad, Mette Hansen, Gøran Paulsen

**Affiliations:** 1 Department for Physical Performance, Norwegian School of Sport Sciences, Oslo, Norway; 2 Mouvement – Interactions – Performance, MIP, UR 4334, Nantes Université, Nantes, France; 3 Institut Universitaire de France, Paris, France; 4 Department for Public Health, Aarhus University, Århus, Denmark

**Keywords:** Hormonal contraception, Birth control pill, Resistance training, Estrogen, Estradiol

## Abstract

**Purpose:**

This study evaluated the effects of eccentric resistance training on hamstring muscles properties in women using oral contraceptives (OC) and naturally menstruating women.

**Methods:**

Before and after the training intervention, we measured maximal isometric and eccentric moment of the knee flexor muscles, thickness and shear wave velocity (SWV) of the biceps femoris long head (BFlh), semitendinosus (ST) and semimembranosus (SM) muscles, and BFlh fascicle length using ultrasonography and dynamometry.

**Results:**

The 12-week training intervention resulted in a modest but statistically significant increase in resting BFlh fascicle length (~2% on average, *p* = 0.005) across both groups, with no observed effect of OC use on any variable. The muscle strength and thickness increased comparably in both groups, with an average increase of ~20% in isometric knee flexor moment and 14% in eccentric knee flexor moment (*p* < 0.001 for both tests). ST muscle thickness increased by ~4% on average (*p* = 0.016), while no main effect of training was observed for SM and BFlh thickness. The shear modulus of the SM muscle decreased by 9% (*p* = 0.021) for the combined groups. However, it remained unchanged in the BFlh and ST muscles, and OC use did not influence these measurements either.

**Conclusion:**

These findings suggest that eccentric training induces similar adaptations in women regardless of OC use. Furthermore, fascicle lengthening was not attributable to changes in muscle shear modulus under the present conditions.

**Supplementary Information:**

The online version contains supplementary material available at 10.1007/s00421-025-05753-x.

## Introduction

Over 150 million women globally use oral contraceptives (OC) for both menstrual regulation and contraception (United Nations Department of Economic and Social Affairs, UN Population Division 2022). The combined oral contraceptive pill, one of the most prevalent types of OC, contains both synthetic estrogen and progestins. This type of OC suppresses the natural hormonal cycle, particularly reducing levels of endogenous estrogen, progesterone, free testosterone, insulin-like growth factor 1, and enhancing cortisol level (Hansen et al. 2011; Zimmerman et al. 2014; Eisenhofer et al. 2017).

Several studies, though not all, suggest that such hormonal alterations may alter the regulation of contractile and connective tissue properties under increased loading. At the cellular level, the consumption of 2nd and 3rd generation OC appear to enhance the myogenic response in women engaged in slow concentric resistance training compared to naturally menstruating women. This is demonstrated by a larger increase in type I fiber cross-sectional area (CSA, Dalgaard et al. 2019) and an increased number of satellite cells in type II fibers (Oxfeldt et al. 2020). In contrast, OC users may exhibit a diminished adaptive response in muscle connective tissue. This is suggested by a lower exercise-induced tendon collagen synthesis in OC users (Hansen et al. 2008) and the absence of an increase in muscle collagen fractional synthesis rate following exercise in OC users (Hansen et al. 2009), although the latter lacks statistical evidence of an interaction effect. These opposing effects of OC on muscle and connective tissues adaptations could be particularly significant in the context of eccentric resistance training in OC users, a subject that has yet to be explored. In addition to improving muscle mechanical function, eccentric resistance training typically results in an increased length of resting muscle fascicles (see Gérard et al. 2020 for review), which may provide protection against muscle strain injuries (Bourne et al. 2018). However, the precise factors that influence resting fascicle length are still not completely understood and have been attributed to one of two mechanisms. The first mechanism involves the addition of sarcomeres in series (Morgan and Talbot 2002; Franchi et al. 2017), which was recently demonstrated in humans (Andrews et al. 2024). However, a second—non-exclusive—mechanism has been proposed, suggesting that sarcomere lengthening could also be due to increased compliance of sarcomeric proteins and/or connective tissues (Pincheira et al. 2022). Albeit not demonstrated, this theory may align with the frequently observed increase in joint flexibility (Diong et al. 2022) and the apparent decrease in muscle stiffness (Mahieu et al. 2008) associated with eccentric training.

Interestingly, either theories would favor fascicle lengthening in women consuming OC, either via the potentiated myogenic response or a reduced collagen synthesis. The purpose of the present study was to compare the effects of eccentric resistance training on muscle fascicle length and shear modulus in OC users and in eumenorrheic non-users. We hypothesized that the training intervention would increase biceps femoris (long head, BFlh) fascicle length in both groups, but that a greater training-induced fascicle lengthening would be observed in OC users. This muscle was chosen because previous research has shown that it can be assessed reliably and since, relative to ST and SM, it may experience greater strain during strenuous activities (e.g., sprinting, Schache et al. 2012) and is particularly prone to injuries (Dimmick and Linklater 2017). Additionally, considering the hypothesis of increased tissue compliance after eccentric resistance training (Pincheira et al. 2022), we also hypothesized that the shear modulus of the BFlh and other hamstring muscles would decrease post-training, with a more pronounced effect in OC users.

## Methods

### Participants

Thirty-nine women were recruited via advertisement and social media. The participants were carefully selected based on an initial questionnaire to assess inclusion and exclusion criteria. All participants were required to be in the age range of 18–40 years, to not have taken part in regular strength training (≤1 strength session per week) in the past 6 months prior to the intervention, and to be free of injury or condition preventing resistance training. Users of oral contraceptives (OC, *n* = 20) were included if they had been using combined OC pills (as opposed to progestogen-only pills) of the 2nd or 3rd generations for at least 6 months prior to the intervention, and no other form of hormonal contraception. Participants not using OC (NOC, *n* = 19) were included if they had not used any hormonal birth control in the past 6 months, had a menstrual cycle within a typical range of 28–32 days, and were not undergoing pregnancy. All participants signed a written informed consent. The study protocol was approved by the institutional ethics committee (No. 24-260917) and conducted in accordance with the Declaration of Helsinki.

### Experimental design

The study was part of a larger project investigating the influence of OC on training outcomes, which involved different muscle groups and modalities of resistance training. However, these aspects did not interfere with the present training protocol, which focused on hamstring muscles. The training period lasted for approximately 12 weeks, corresponding to three menstrual cycles for the NOC group or to three pill cycles for the OC group. The tracking of menstrual and pill cycles was done using a smartphone application (Clue, Biowink, GmbH) and ovulation tests (Babyplan, BPL Diagnostics As, Oslo, Norway) in NOC participants. All participants trained twice a week, with supervision provided for the first two sessions and at least two additional sessions over the course of the training period.

**Fig. 1:**
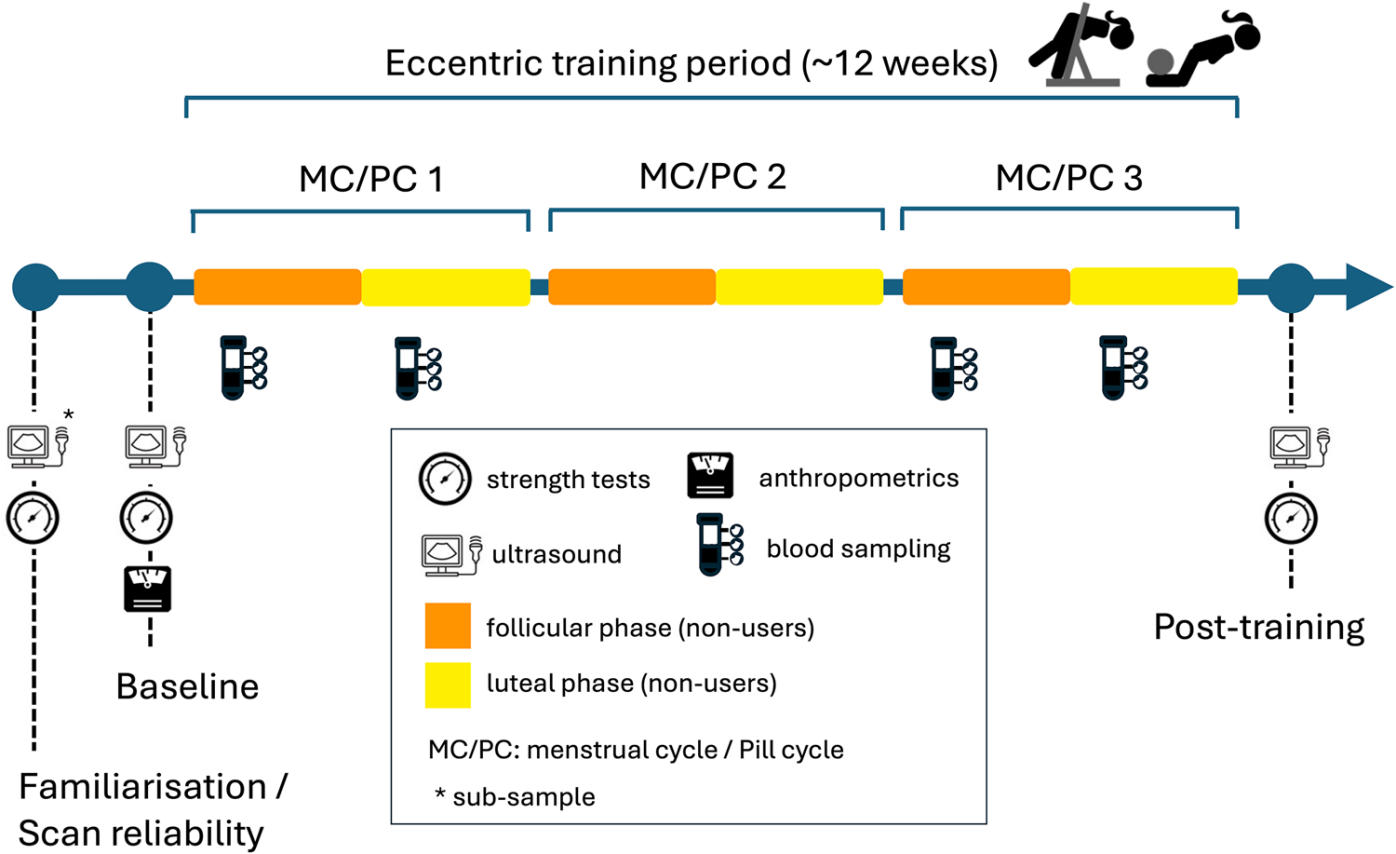
Experimental design

Participants visited the lab on two occasions before the onset of training and once after the training period (Fig. 1). The initial visit was dedicated to collecting data from a subset of participants (*n* = 8) to assess the inter-day reliability of ultrasound-based measurements, and to familiarize (all) the participants with strength tests. All main outcome variables were measured during the baseline and the post-training sessions (2nd and 3rd visits respectively). All measurements were performed on the right leg.

### Eccentric resistance training

The study incorporated eccentric knee extension and hip flexion exercises to train the primary functions of the hamstrings, as three of the four hamstring muscles are bi-articular and serve as powerful hip extensors. These exercises were included since each of them was found to induce fascicle lengthening in the biceps femoris long head (Bourne et al. 2017). For the Nordic hamstring exercises, the ankles of the participants were held in place to the ground with wall fixtures or with loaded barbells and their knees slightly elevated on a ~15-cm pad. The load progression was primarily regulated with a resistance band, whereby loading was increased by using bands with a lower stiffness or by removing the band entirely. This assistance and the knee elevation allowed participants to complete Nordic hamstring exercises over the full range of motion (i.e., up to 0° of knee flexion). Participants progressing to a sufficient strength level to perform the exercises without any assistance were instructed to increase the number of repetitions per set. For the single-leg eccentric hip flexions, a Roman chair was used to secure the ankle of the working leg in position. Load progression was achieved by having participants hold a weight either by their chest or over their heads. The number of repetitions was guided by the rate of perceived exertion (RPE), with failure subjectively assessed by either the training instructor or the participants themselves. We used repetition in reserve (RIR) and RPE-based training to ensure all participants exerted the same level of effort. Resistance training based on RIR has been demonstrated to be an effective way to autoregulate resistance training, allowing for individual adjustments based on daily readiness and performance levels (Larsen et al. 2021). Progress was also assessed by either increasing the load or total number of repetitions performed weekly within each menstrual/OC cycle (Table 1). Furthermore, two sets of Nordic hamstring exercises and eccentric hip flexions exercises were added to increase the overall training volume, to optimize gains in knee flexor strength and fascicle length (Severo-Silveira et al. 2021).

**Table 1:** Resistance training program

Weekly session	Exercise	MC/PC 1	MC/PC 2	MC/PC 3
1st	Nordic hamstrings	2 × 10–12	2 × 6–10	3 × 5–8
1st	Eccentric hip flexion	2 × 8–12	3 × 6–10	3 × 5–8
2nd	Nordic hamstrings	2 × 10–12	3 × 6–10	3 × 5–8
2nd	Eccentric hip flexion	2 × 8–12	2 × 6–10	3 × 5–8

*MC* menstrual cycle, *PC* pill cycle

### Serum hormones and monitoring of menstrual cycles

Serum estradiol, progesterone, follicle-stimulating hormone (FSH) and luteinizing hormone (LH) were measured in all participants, at time points corresponding to the follicular phases (FP) and luteal phases (LP), during the 1st and 3rd menstrual cycles (Fig. 1). To standardize sampling to the diurnal peak of circulating estrogen (Bao et al. 2003), fasted blood was drawn from an antecubital vein in the early follicular phase, 3–6 days after the onset of menstruation (NOC), or 3–6 days in the withdrawal phase (OC), and in the mid-luteal phase, 20–24 after the onset of menstruation (NOC) or after the active pill phase, (OC) of the first and third cycles of training. Blood samples were first centrifuged and stored at +4 °C and sent to a private laboratory where serum levels of target hormones were measured using immunochemiluminometric assays (ADVIA Centaur XPT Immunoassay System, Siemens Healthineers) within 8 h. The analytical coefficients of variation for estradiol, progesterone, FSH, LH and SHBG were 7.9, 3.5, 3.1, 2.4 and 3.8%, respectively (values provided by the analysis laboratory). The detection thresholds were 0.04 nmol/L, 3.00 nmol/L, 0.3 IU/L, and 0.07 IU/L, respectively, for estradiol, progesterone, FSH and LH.

### Muscle morphology and shear modulus

**Fig. 2:**
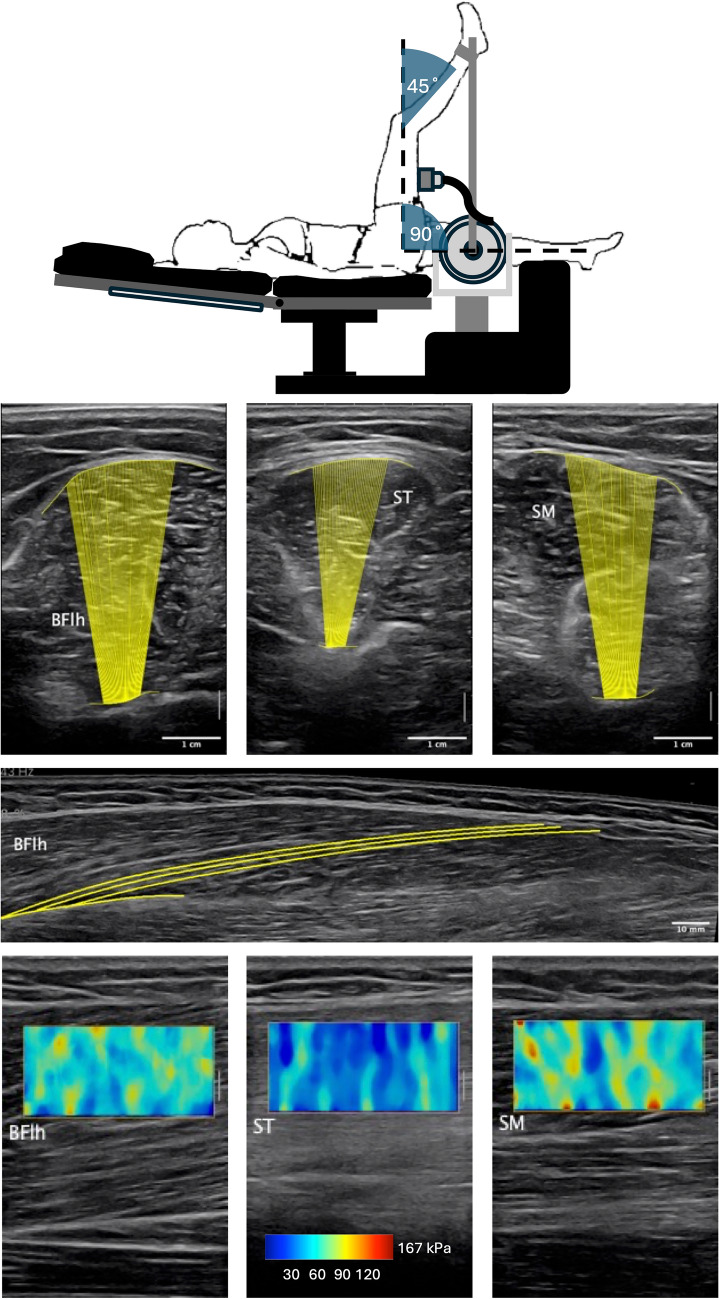
Experimental setup and representative ultrasound scans collected for the measurement of muscle thickness (1st row), fascicle length (2nd row) and muscle shear modulus (3rd row). BFlh: biceps femoris long head muscle, ST: semitendinosus muscle, SM: semimembranosus muscle

For the ultrasound-based measurements (Fig. 2), the participants were positioned supine on the backrest of a dynamometer chair. Their right leg was elevated and secured on the dynamometer attachment. The hip and knee angles were set at 90˚ and 45˚ respectively, with 0˚ corresponding to full extension in both cases. This scanning position imposed a passive tension on the hamstrings, and was chosen as it may be more sensitive to inter-individual differences in shear wave-based measurements (Avrillon et al. 2020) and to acute changes in this variable (Lacourpaille et al. 2017) compared to measurements taken in slack muscles. To identify scanning areas relative to mid-femur length, the femur length was measured between the greater trochanter and the lateral epicondyle. Additionally, matching the scanning areas in subsequent sessions was ascertained by visually matching echoic features (pattern of connective tissue, blood vessels) from initial scans. All ultrasound scans requiring manual analysis were anonymized.

Muscle thickness and fascicle length were measured using B mode ultrasound (L10-2/38 mm, Mach 30, Hologic SuperSonic Imagine, Aix-en-Provence, France). Transversal scans of the biceps femoris long head (BF), semitendinosus (ST), and semimembranosus (SM) muscles were collected at 50% of femur length to measure thickness. Muscle thickness was obtained offline by manually outlining superficial and deeper aponeuroses and by measuring the mean distance between these structures (Fiji ImageJ distribution, Schindelin et al. 2012). While muscle thickness does not capture the same information as the volume or cross-sectional area in terms of force production capacity, it is a sensitive marker of hypertrophy (Franchi et al. 2018) and pilot testing suggested that it would be measured more reliably for this population. Muscle thickness measured from two different days in a sub-sample of participants (*n* = 8) indicated moderate to good (Koo and Li 2016) reliability, with a 95% intraclass correlation coefficient (ICC(3, 1)) of 0.81, 0.88 and 0.70, for the BFlh, ST and SM muscles, respectively. The raw typical error (TE) was 1.5, 1.2 and 2.3 mm and the coefficient of variation (CV) was 3.4, 4.6 and 4.9% respectively, for the BFlh, ST and SM muscles. The minimum detectable change at 95% confidence interval (MDC), computed as standard error of measurement × √2 × 1.96, was 4.2, 3.3 and 6.5 mm, respectively, for the BFlh, ST and SM muscles.

Architecture scans were collected from the BF muscle. With an initial probe position at approximately 7-cm proximal to the femur mid-line, the probe was moved distally over the muscle to collect a panoramic scan of complete fascicles. Fascicle length was measured offline (Fiji ImageJ distribution). In each image, three fascicles were manually drawn between aponeuroses by following regional gradients of visible segments. The resultant segmented lines were smoothed with a spline fitting and the mean length of the curves was retained as the final value. The fascicle length of the BFlh measured from two different days in a sub-sample of participants indicated an excellent (Koo and Li 2016) reliability, with an ICC(3, 1) of 0.91, a TE of 0.4 mm, an MDC of 1 mm, and a CV of 1.8%.

Shear wave elastography scans were collected from BFlh, ST and SM muscles with the same equipment as for B mode scans (presets: penetration mode, smoothing level 5, persistence off, sampling rate: 1 Hz). The scans were acquired with the probe orientated along the longitudinal axis of the muscles, at 50% of femur length. Ten frames were sampled for each muscle, and shear wave color maps were analyzed offline using a custom script (Python, swepy, Seynnes 2023). Shear wave data were averaged across frames and used as an indicator of instantaneous material property, by computing shear modulus from wave velocity data (Hug et al. 2015). To avoid bias due to pixel saturation, trials in which saturated pixels (≥500 kPa) exceeded 5% (averaged across frames) were excluded. Muscle shear modulus measured from two different days in a sub-sample of participants (*n* = 7) indicated good (Koo and Li 2016) reliability for the BFlh and SM muscles, with ICC(3, 1) coefficients of 0.89 and 0.86, respectively, a TE of 4.0 and 4.6 kPa, an MDC of 11.0 and 12.8 kPa and a CV of 6.7 and 8.5%, respectively. The ICC for the ST muscle was however moderate (coefficient = 0.73), with higher TE (7.6 kPa), MDC (21.0 kPa) and CV (21.2%).

### Isometric and eccentric moment of the knee flexor muscles

To monitor changes in maximal strength of the hamstring muscles, we measured the knee flexor moment resulting from isometric maximal voluntary contraction (MVC) and from eccentric MVC (Humac Norm, Computer Sports Medicine Inc.; Massachusetts, USA). The tests were performed in a seated position with the back angle set at 70° of flexion (0° being when the hip is fully extended). Each test started with a specific warm-up consisting of 10 sub-maximal contractions. For the isometric test, the knee joint angle was set at 50°. The eccentric tests were performed over a knee range of motion of 10°–90° (0° being when the knee is fully extended), at a velocity of 30°/s. Each test was attempted three times. However, if any attempt yielded a result differing by more than 10% of the moment from the best attempt, the attempt was repeated. Each test/attempt was separated by a 2-min rest period.

### Statistical analysis

Differences between groups at baseline were tested with a paired Student’s *t* test. Between-group differences in cycle-averaged hormonal levels were tested with a Mann–Whitney *U* test, as these variables were not normally distributed (as indicated with a Shapiro–Wilk test). For all main outcome variables, a repeated mixed-factor ANOVA (with training and group as factors) was used to determine whether the training intervention resulted in significant changes and whether there was an interaction with OC consumption. The effect sizes were reported as partial eta squared ($\eta_{{\text{p}}}^{2}$). When a significant interaction effect was found, a post hoc comparison was conducted using the Bonferroni correction to control for Type I error and the effect size was measured as the Cohen’s *d*. The analyses were performed in JASP 0.19.0 (2024). The *α* level was set at *p* < 0.05 and data are reported as mean ± standard deviation.

## Results

During the training intervention, six participants dropped out for reasons unrelated to the intervention itself and one NOC participant was excluded because of her menstrual cycle being too long (Janse de Jonge et al. 2019). The study was therefore completed by 32 women, including 17 in the NOC group and 15 in the OC group. Both groups completed a similar number of training sessions (23.5 ± 1.1 vs. 23.5 ± 1.3 sessions, respectively, for the OC and the NOC groups, *p* = 0.885). Most OC users consumed 2nd generation of OC (*n* = 13). All consumed OC containing 20 or 30 μg of ethinylestradiol and various progestins (levonorgestrel, *n* = 13, desogestrel *n* = 2). The characteristics of both groups were comparable, although they differed on average by 3 years in age (27.9 ± 2.8 vs. 31.6 ± 4.8 years, respectively, for OC and NOC groups, *p* = 0.014). The average height was 171.4 ± 5.6 cm for OC users and 170.2 ± 8 cm for non-OC users (*p* = 0.616). The average weight was 70.0 ± 14.7 kg for OC users and 69.3 ± 9 kg for non-OC users (*p* = 0.871). The body mass index (BMI) was 23.8 ± 4.7 kg/m^2^ for OC users and 24.0 ± 2.9 kg/m^2^ for non-OC users (*p* = 0.898). The duration of OC use among users was 8.1 ± 4.6 years.

Since no prior study had reported the effect of eccentric training in women using oral contraceptive, the target sample size was based on an a priori power calculation from the changes in muscle cross-sectional area induced by conventional resistance training in OC-users and non-users, as previously reported by Dalgaard et al. (2019), 16 per group. A posteriori calculation based on the fascicle length measurements revealed that—despite the dropouts—the power of the study was 0.83, which is above the commonly accepted threshold for similar studies.

### Serum hormones

The results from the serum hormones analysis are also reported in the other articles (unpublished at the time of submission) arising from the same research project (see experimental design). The hormone concentration data confirmed the anticipated differences between the OC users and non-users groups (Table 2; Supplementary material). Mean values (corresponding to a whole menstrual/pill cycle) of estradiol (*p* < 0.001), FSH (*p* = 0.020) and LH (*p* < 0.001) were lower in OC users compared to NOC participants, while sex hormone binding globulin levels were higher (*p* < 0.001). Progesterone was undetectable (detection level = 3 nmol/L) in NOC participants during FP and in OC users at any time point within the pill cycle.

**Table 2:** Serum hormonal levels of sex hormones

	OC-users (*n* = 15)			Non-OC (*n* = 17)			*p* value
	CP	WP	Cycle mean	FP	LP	Cycle mean	
Estradiol (nmol/L)	0.09 ± 0.04	0.07 ± 0.01	0.08 ± 0.02	0.35 ± 0.30	0.52 ± 0.22	0.44 ± 0.19	<0.001
Progesterone (nmol/L)	<3.0	<3.0	n/a	<3.0	27.7 ± 13.2	n/a	n/a
FSH (IU/L)	5.6 ± 3.7	2.8 ± 2.5	4.2 ± 2.6	8.4 ± 2.8	4.8 ± 1.7	6.6 ± 1.9	0.020
LH (IU/L)	2.7 ± 1.8	2.5 ± 3.2	2.6 ± 2.0	7.9 ± 10.0	5.4 ± 3.0	6.6 ± 5.1	<0.001
SHBG (nmol/L)	110.6 ± 33.1	122.8 ± 34.1	116.7 ± 32.7	54.9 ± 20.6	59.7 ± 21.8	57.3 ± 21.1	<0.001

*OC* oral contraceptives, *CP* active pill consumption phase, *WP* pill withdrawal phase, *FP* follicular phase, *LP* luteal phase, *FSH* follicle-stimulating hormone, *LH* luteinizing hormone, *SHBG* sex hormone binding globulin

The *p* values result from a Mann–Whitney *U* test performed on cycle-mean values

### Isometric and eccentric knee flexor moment

**Fig. 3:**
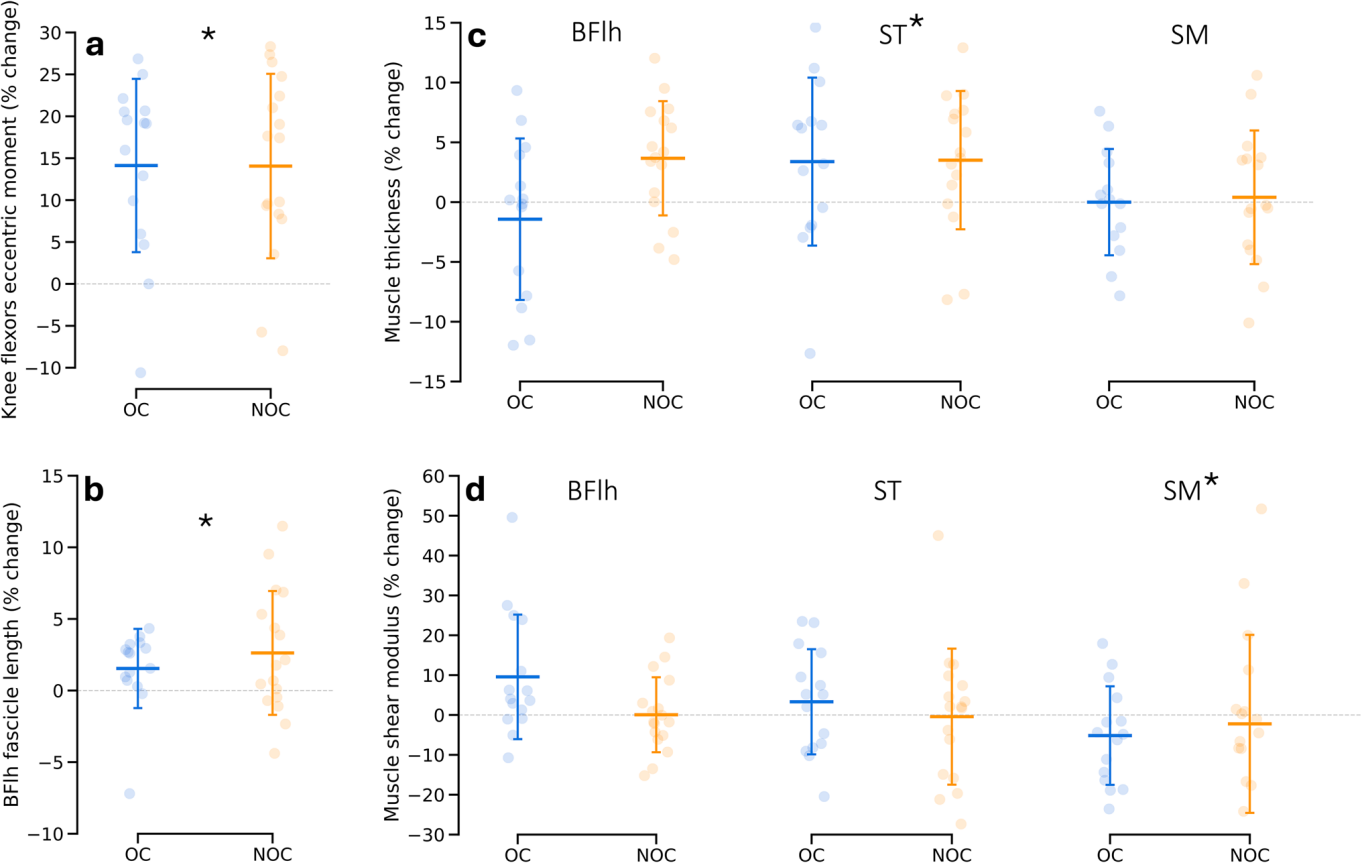
Changes in knee flexors eccentric moment (**a**, *n* = 32), fascicle length of the biceps femoris long head muscle (**b**, *n* = 32), thickness (**c**, *n* = 31) and shear modulus (**d**, *n* = 28). OC: oral contraceptives users, NOC: non-oral contraceptives users, BFlh: biceps femoris long head muscle, ST: semitendinosus muscle, SM: semimembranosus muscle. * *p* < 0.05, main effect of training

Strength increased in all participants (Table 3). The main effect of training was significant for the knee flexor isometric moment (+20.2 ± 13.6% and +19.2 ± 13.4%, respectively, for OC and NOC groups, *p* < 0.001) and the eccentric moment (+14.1 ± 10.3% and +14.1 ± 11.0%, respectively, for OC and NOC groups, *p* < 0.001, Fig. 3). However, we did not observe any interaction effect between training and OC consumption (*p* = 0.974 and *p* = 0.831, respectively, for the isometric and eccentric tests).

**Table 3:** Dynamometry and ultrasound measurements outcomes before and after the training intervention

	OC group			NOC group			Training main effect		Group × training interaction	
	*n*	Pre	Post	*n*	Pre	Post	*p* values	$\eta_{{\text{p}}}^{2}$	*p* values	$\eta_{{\text{p}}}^{2}$
KF isometric moment (N m)	15	75.5 ± 13.9	89.9 ± 15.0	17	78.9 ± 13.2	93.2 ± 13.4	**<0.001**	0.729	0.974	3.672 × 10^−5^
KF eccentric moment (N m)	15	113.6 ± 23.8	128.7 ± 25.5	17	119.0 ± 17.6	135.1 ± 20.3	**<0.001**	0.609	0.831	0.002
BFlh fascicle length (mm)	15	144.3 ± 15.9	146.3 ± 14.9	17	149.6 ± 12.7	153.6 ± 15.2	**0.005**	0.236	0.341	0.030
BFlh thickness (cm)	15	35.7 ± 4.4	35.1 ± 3.8	16	34.5 ± 2.9	35.7 ± 2.9	0.396	0.025	**0.017**	0.181
ST thickness (cm)	15	34.6 ± 4.7	35.4 ± 4.6	16	33.5 ± 3.3	34.6 ± 3.2	**0.016**	0.183	0.742	0.004
SM thickness (cm)	15	40.3 ± 4.5	40.1 ± 3.7	16	38.8 ± 5.0	38.9 ± 4.5	0.797	0.002	0.717	0.005
BF SWM (kPa)	12	50.6 ± 14.6	53.1 ± 11.3	16	51.7 ± 9.3	51.7 ± 11.2	0.499	0.018	0.484	0.019
ST SWM (kPa)	12	34.9 ± 13.2	37.1 ± 10.9	16	34.9 ± 11.6	32.6 ± 8.6	0.987	9.961 × 10^−6^	0.245	0.052
SM SWM (kPa)	12	53.1 ± 18.0	44.7 ± 11.0	16	47.8 ± 14.1	43.2 ± 17.1	**0.021**	0.189	0.466	0.021

The bold values denote significant effects

*OC* oral contraceptives, *KF* knee flexor, *BFlh* biceps femoris long head muscle, *ST* semitendinosus muscle, *SM* semimembranosus muscle, *SWM* shear wave modulus

### Muscle thickness

Muscle thickness data from one participant in the NOC group were not collected as the result of a logistical error. The analysis was, therefore, performed on 16 NOC participants and 15 OC participants. The thickness of the ST muscle increased in combined groups (main training effect, *p* = 0.016) but the thickness of the BFlh and SM muscles did not change significantly (no main effect of training, Table 3; Fig. 3). The interaction effect between training and OC consumption reached significance for the BFlh muscle (*p* = 0.017). However, the post-hoc analysis did not reveal any significant changes within each group (*p* = 0.127, Cohen’s *d* = −0.343 and *p* = 1.000, Cohen’s *d* = 0.169, respectively, for the NOC and OC groups). No significant interaction effect between training and OC consumption was found for the ST and SM muscles.

### Muscle fascicle length

The fascicles of the BFlh muscle were longer in combined groups after training (main training effect, *p* = 0.005), but there was no interaction between training and OC consumption (Table 3; Fig. 3).

### Muscle shear modulus

The data from four participants were excluded because of excessive saturation (see exclusion criterion in the “Methods” section). The results of the shear modulus analysis are, therefore, based on 12 OC participants and 16 NOC participants. A main effect of training was found for the shear modulus of the SM muscle (−10.1 ± 24.1% for the OC group and −7.8 ± 35.1% for the NOC group, *p* = 0.021) but not for the BFlh and ST muscles (Table 3; Fig. 3). We did not observe any interaction effect between training and OC consumption (*p* = 0.484, *p* = 0.245 and *p* = 0.466, respectively, for the BFlh, ST and SM muscles).

## Discussion

The eccentric training intervention implemented in the present study led to a modest yet statistically significant increase in resting fascicle length (approximately 2% on average) of the BFlh muscle. However, contrary to our hypothesis, we did not observe any effect of OC use on the shear modulus of this muscle or on any of the measured variables in ST and SM. These findings suggest that 12 weeks of eccentric resistance training induces similar adaptations in OC and NOC women. Additionally, the lack of change in resting BFlh shear modulus despite an increase in fascicle length indicates that the latter cannot be attributed to the former, regardless of OC use.

### Muscle strength and thickness

The present training intervention induced comparable increases in strength as seen in other intervention studies on males and females (e.g., Ribeiro-Alvares et al. 2018), with an average increase of ~20% in isometric knee flexor moment and 14% in eccentric knee flexor moment across both groups. Accordingly, we also observed a training impact on the ST muscle thickness (+4% on average), though no main effect of training was seen for the SM and BFlh thickness. An interaction effect (training × group) was however significant for the BFlh thickness (−1.3 ± 6.5% in OC users vs. +3.7 ± 4.8% in non-users, Fig. 3) while it was not for the ST and SM thickness or for strength measurements. Given the lack of main training effect and the non-significance of within-group changes in BFlh thickness (post hoc analysis), we suspect a type I error for this measurement. The exclusive increase in ST thickness in combined groups corroborates recent findings (Frouin et al. 2024) and is consistent with the primary role that this muscle may have for load-bearing function (Evangelidis et al. 2021). Previous studies—yet not all (e.g., Severo-Silveira et al. 2021)—align with the present lack of increase in BFlh thickness in the OC group, showing no thickness change in response to eccentric resistance training despite strength enhancements (Timmins et al. 2016; Ribeiro-Alvares et al. 2018). Although we did not measure pennation angle, the reduction in this parameter observed in some eccentric training studies (Ribeiro-Alvares et al. 2018) may mitigate the increase in thickness, despite a lengthening in fascicles. Additionally, the differences in thickness changes between studies could also be attributed to measurement methods (e.g., longitudinal image vs. transverse image, specific location within the muscle where the measurement was taken).

### Fascicle length

Fascicle length measured in this study (range 12.3–18 cm) appears larger than reported in some previous studies (e.g., Wiesinger et al. 2021; Pincheira et al. 2022), including studies using panoramic scans (Seymore et al. 2017; Marušič et al. 2020). However, our figures of fascicle length are consistent with the hip and knee joint configuration used in this study (Chleboun et al. 2001). The percent increase in fascicle length was similar in both groups, albeit smaller than previously reported (+12–21%, Bourne et al. 2017; Pincheira et al. 2022). Prior research examining the effects of eccentric training on muscle architecture in women is scarce. One study on the vastus lateralis muscle showed no significant sex-related difference, despite a 7% increase in women, which is markedly lower than typically reported (Coratella et al. 2018). The 2% increase we observed, derived from a robust methodology including blinding and segmentation in panoramic scans, suggests that this experimental objective was achieved, albeit modest in magnitude. However, it is noteworthy that this increase in fascicle is near the detection threshold, making it more challenging to reveal potential differences between groups. Contrary to our hypothesis, we did not observe greater fascicle lengthening in OC users. In contrast, some recent studies suggest a heightened myogenic response in OC users (Dalgaard et al. 2019; Oxfeldt et al. 2020). Although the general assumption is that OC may overall favor a myogenic response due to the greater amount of circulating estrogen, the impact of OC on other hormones may affect the anabolic response in more complex ways. For instance, this is suggested by the higher SHBG levels observed in OC users (Table 2), which reflect the reduction of free testosterone levels induced by OC (Zimmerman et al. 2014). This reduction may consequently decrease the anabolic effect on collagen metabolism. On the other hand, the progestins contained in the OC of the participants of this study are categorized as androgenic, with their affinity for androgen receptors potentially leading to anabolic effects. A greater myogenic response could theoretically contribute to the addition of serial sarcomeres (Andrews et al. 2024) and promote further fascicle lengthening. However, such an influence on the current fascicle lengthening was not observed. If sarcomerogenesis occurred in the present study, the lack of statistical differences between groups does not support this process as a determinant for the hypothesized greater fascicle lengthening in OC users. Moreover, this lack of group difference also challenges our postulated non-myogenic mechanism of reduced muscle stiffness.

### Muscle shear modulus

We observed a ~9% decrease in the shear modulus of the SM muscle, but no statistically significant difference was found for the ST or BFlh muscles, where fascicle length was measured. These findings are consistent with those of Kawama et al., who found a similar pattern of change in hamstrings shear modulus in males following eccentric stiff-leg deadlift training (Kawama et al. 2024). Of note, a previous study examining hamstrings mechanical properties found lower SM shear modulus in several classes of athletes compared to controls, while values were similar for the BFlh and ST muscles (Avrillon et al. 2020). These observations align with the differential hypertrophy noted in this study, albeit in a single muscle region, and the notion that synergist muscles may fulfil specific roles related to loading requirements (Kopydlowski et al. 2014). Previous studies using SWE to investigate the effects of eccentric training on BFlh material properties in males and females found no change (Seymore et al. 2017; Vatovec et al. 2021). Our protocol yielded slightly different results, possibly due to scanning the muscles at a greater length than in previous studies, which may have enhanced the sensitivity of SW measurements. However, the lack of change in shear modulus for the BFlh should be interpreted with caution. The sensitivity threshold of SWE measurements may have been insufficient to detect subtle training-induced variations or within-group differences, particularly if these variations corresponded with the—modest—changes in fascicle length as hypothesized. A posteriori power calculations indicate that our SWE measurements may have been under-powered (0.41, 0.39 and 0.75, for the BFlh, ST and SM muscles, respectively). Although our findings do not support any influence of combined OC on adaptations in muscle shear modulus with eccentric training, the relationship between this variable and fascicle length should be re-examined with an intervention including more participants and/or causing greater fascicle lengthening.

### Strengths and limitations

This study addresses several unanswered questions regarding skeletal muscle adaptability in women. To our knowledge, this is the first study to report the effects of eccentric resistance training on BFlh fascicle length and investigate the impact of combined OC consumption on adaptations to this training type. However, we recognize some study limitations. In an effort to include OC users with a similar hormonal profile, we primarily recruited women using 2nd generation OC of the same type (*n* = 11 out of 15). We resolved to recruit two more participants using 2nd-generation OC with a different composition and two users of 3rd-generation OC to complete recruitment. Although the composition of OC differed in these four participants, they also contained 20–30 µg of ethinyl estradiol and the same progestin (levonorgestrel, *n* = 2) or a progestin with a lower androgenicity (desogestrel, *n* = 2). In addition, performing the statistical analysis excluding these participants did not reveal any interaction effect with OC either. However, considering the influence of ethinyl estradiol dosage observed previously (Dalgaard et al. 2019) and the possible influence of progestin androgenicity (Burrows and Peters 2007) on hypertrophy, future research should strive to recruit a more homogeneous sample regarding the type of OC-use. Some aspects related to training and testing should also be acknowledged as potential limitations. Training monitoring was based on self-reports. Although this method is less ideal than supervising all training sessions, it was chosen for logistical reasons to provide participants with sufficient scheduling flexibility and to prevent attrition. The fact that both groups experienced similar strength gains, which were comparable in magnitude to those reported in previous studies, indicates that the training intervention was effective. Further, we standardized testing timing during the menstrual/OC cycle to account for hormonal variations (McNulty et al. 2020) to a 3–9-day window. Despite this precaution, this time window might have placed some participants in the early consumption or mid-follicular phase, potentially altering hormonal environments. Shear wave elastography measurements are sensitive to small variations in muscular activity (Le Sant et al. 2019). It was logistically not possible to monitor the electromyographic activity of the muscles during scanning in this study and variations in muscle activity may have contributed to the observed variability in shear modulus. Finally, we conducted our measurements of BFlh fascicle length while the participants were in a position that stretched their hamstring muscles. While measuring the architecture of the ST reliably remains challenging, it would have been interesting to assess the architecture of the SM, considering the present results and its important contribution to produce mechanical work. The testing position was selected with the intent to replicate the position in which SWE data were collected. However, the potential mitigating influence of changes in tendon stiffness (Wiesinger et al. 2015) cannot be dismissed.

## Conclusion

A 12-week regimen of eccentric resistance training targeting the knee flexor muscles led to an increase in BFlh fascicle length among young women. This result was consistent regardless of whether the participants used combined OC or not. These findings imply that the impact of OC, previously documented at the cellular level on muscle and connective tissue, does not have a significant effect on muscular adaptations to eccentric training at the macroscopic level. From a clinical perspective, these results suggest that the ability to gain protective adaptations, such as longer fascicles, against hamstring strain injuries following resistance training may not differ between women who use combined oral contraceptives and those who do not.

## Supplementary Information

Below is the link to the electronic supplementary material.

Supplementary file1 (DOCX 2324 KB)

## Data Availability

Data will be available upon reasonable request.

## Abbreviations

OC: Oral contraceptives
BFlh: Biceps femoris long head
ST: Semitendinosus
SM: Semimembranosus
NOC: Oral contraceptives non-user
FSH: Follicle-stimulating hormone
LH: Luteinising hormone
FP: Follicular phase
LP: Luteal phase
ICC: Intraclass correlation
MVC: Maximal voluntary contraction
ANOVA: Analysis of variance
