# Supplementary material for: Eccentric training effects on hamstring muscles in oral contraceptive users and naturally menstruating women
Source: Eur J Appl Physiol. 2025 Mar 15;125(8):2133–44. doi: 10.1007/s00421-025-05753-x (PMC12354606; doi:10.1007/s00421-025-05753-x)
Supplement: Supplementary file 1 — Supplementary file1 (DOCX 2324 KB) [file 421_2025_5753_MOESM1_ESM.docx]

**Supplementary Information (SI)**

**Eccentric training effects on hamstring muscles in oral contraceptive users and naturally menstruating women**

Olivier Seynnes^1^(https://orcid.org/0000-0002-1289-246X), Antoine Nordez^2,3^(https://orcid.org/0000-0002-7276-4793), Lilian Lacourpaille^2^(https://orcid.org/0000-0002-9664-8121), Eirik Hesseberg^1^, Ingvild Vesterhus^1^, Ken Fjeldberg^1^, Martin Kvalvik Engstad^1^, Mette Hansen^4^(https://orcid.org/0000-0002-9664-8121), Gøran Paulsen^1^(https://orcid.org/0000-0002-6350-0112).

^1^ Department for Physical Performance, Norwegian School of Sport Sciences, Oslo, Norway

^2^ Nantes Université, Mouvement - Interactions - Performance, MIP, UR 4334, Nantes, France

^3^ Institut Universitaire de France, Paris, France

^4^ Department for Public Health, Aarhus University, Aarhus, Denmark

Corresponding author: Olivier Seynnes, oliviers@nih.no

Fig. SI 1. Flowchart diagram depicting the recruitment process. OC: oral contraceptives users, NOC: non-oral contraceptives users.


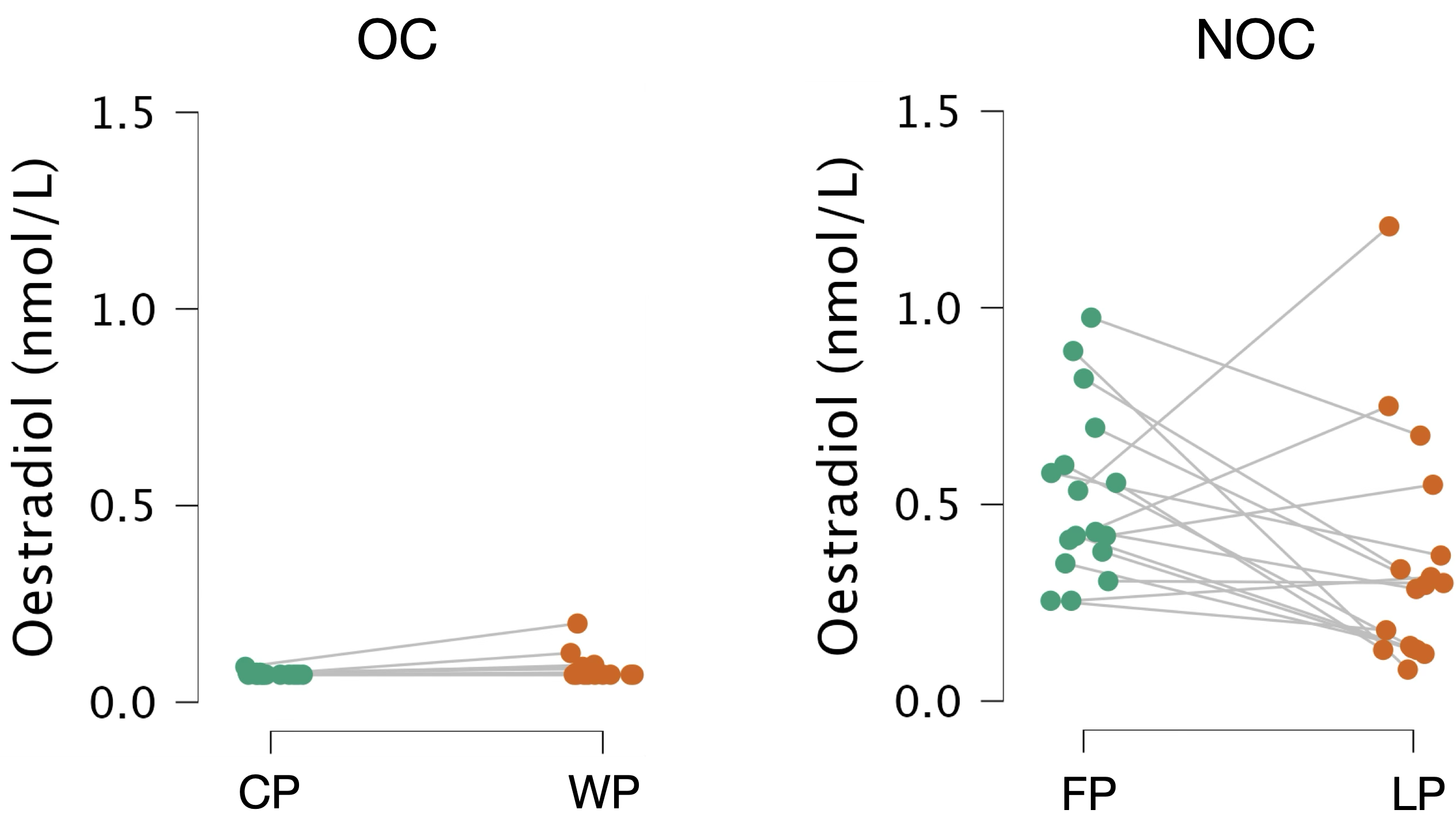


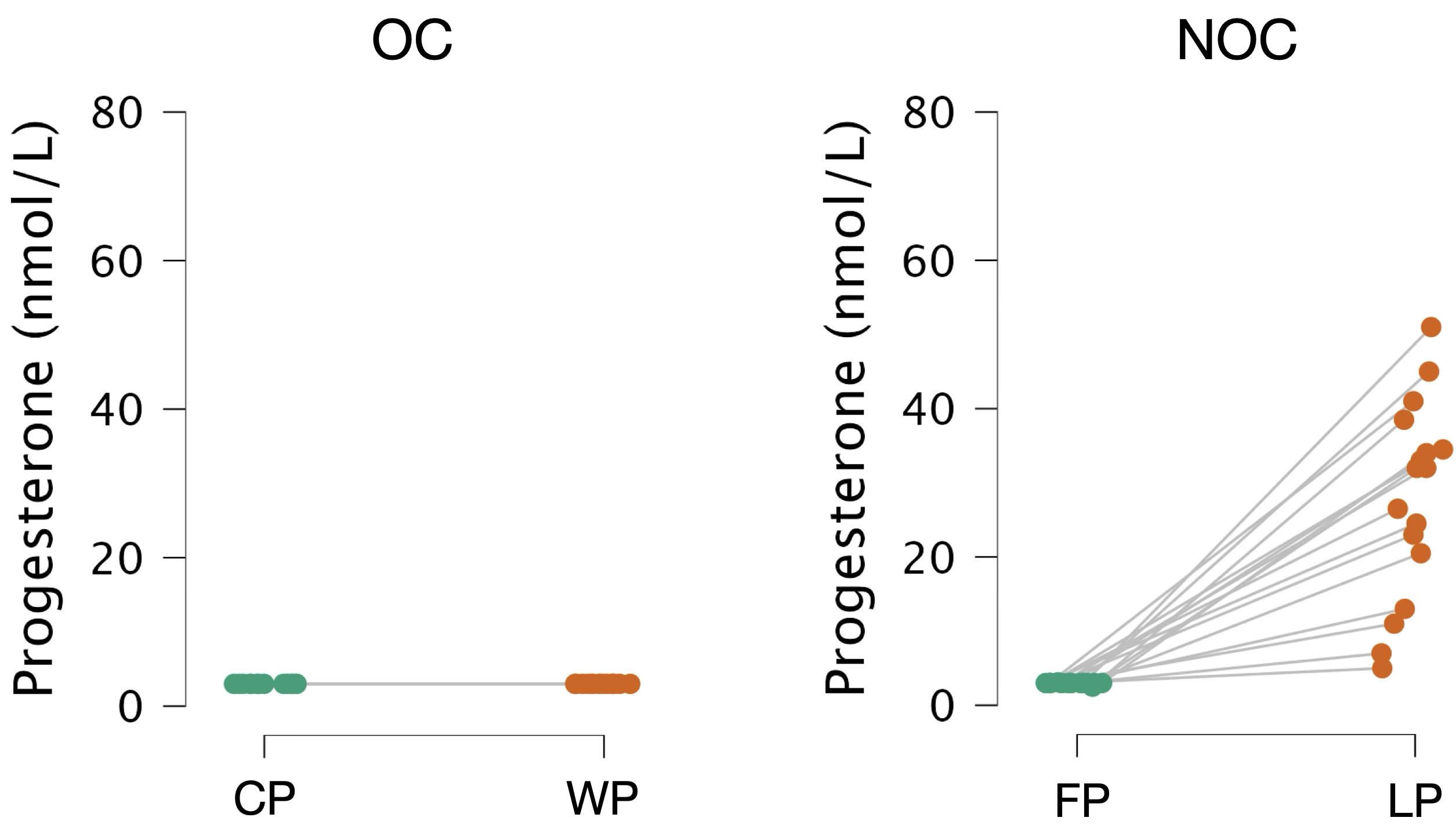


Fig. SI 2. Inter-individual variations in serum hormonal levels of oestradiol and progesterone. OC: oral contraceptives users, NOC: non-oral contraceptives users, CP: active pill consumption phase, WP: pill withdrawal phase, FP: follicular phase, LP: luteal phase.


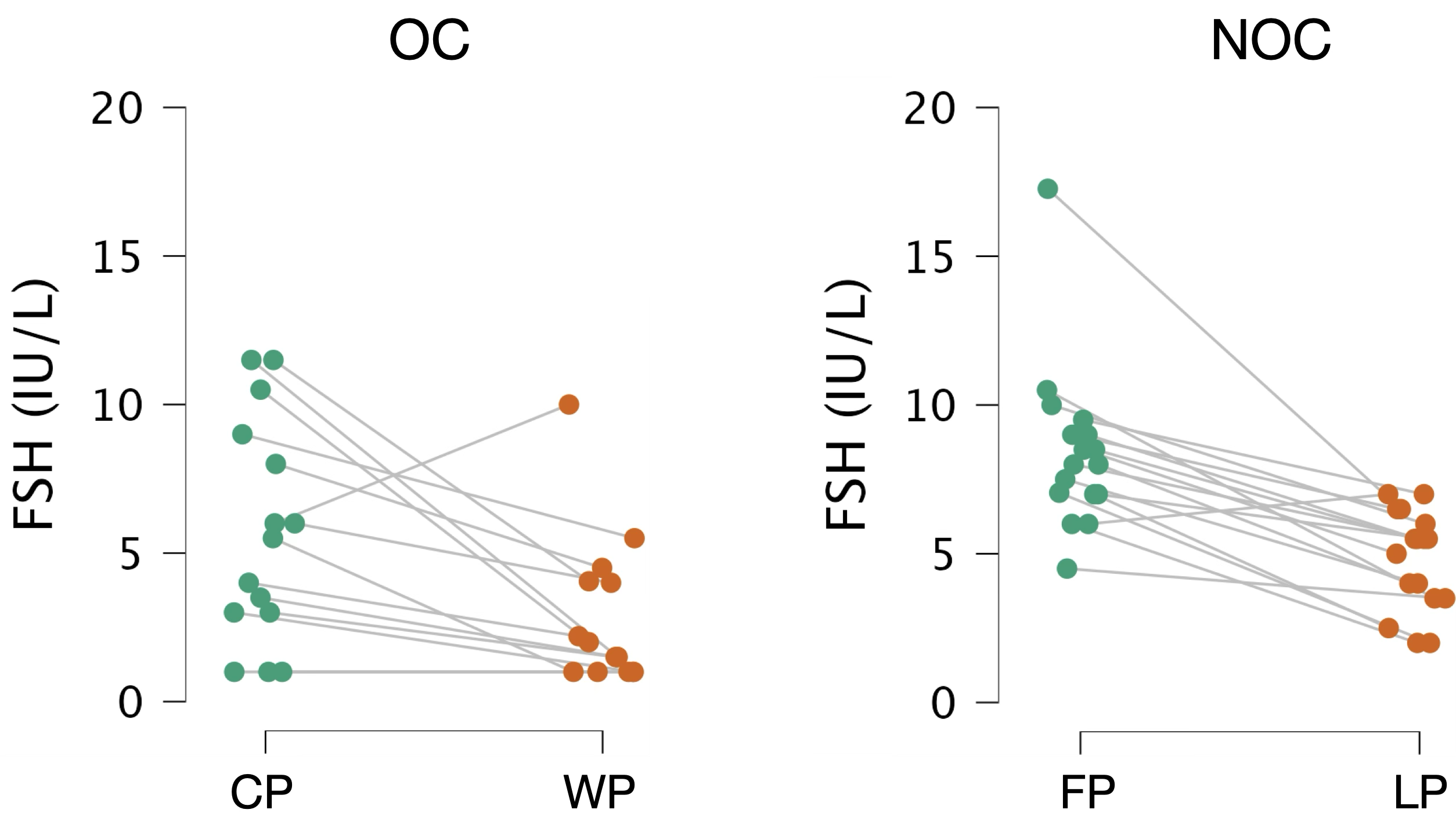


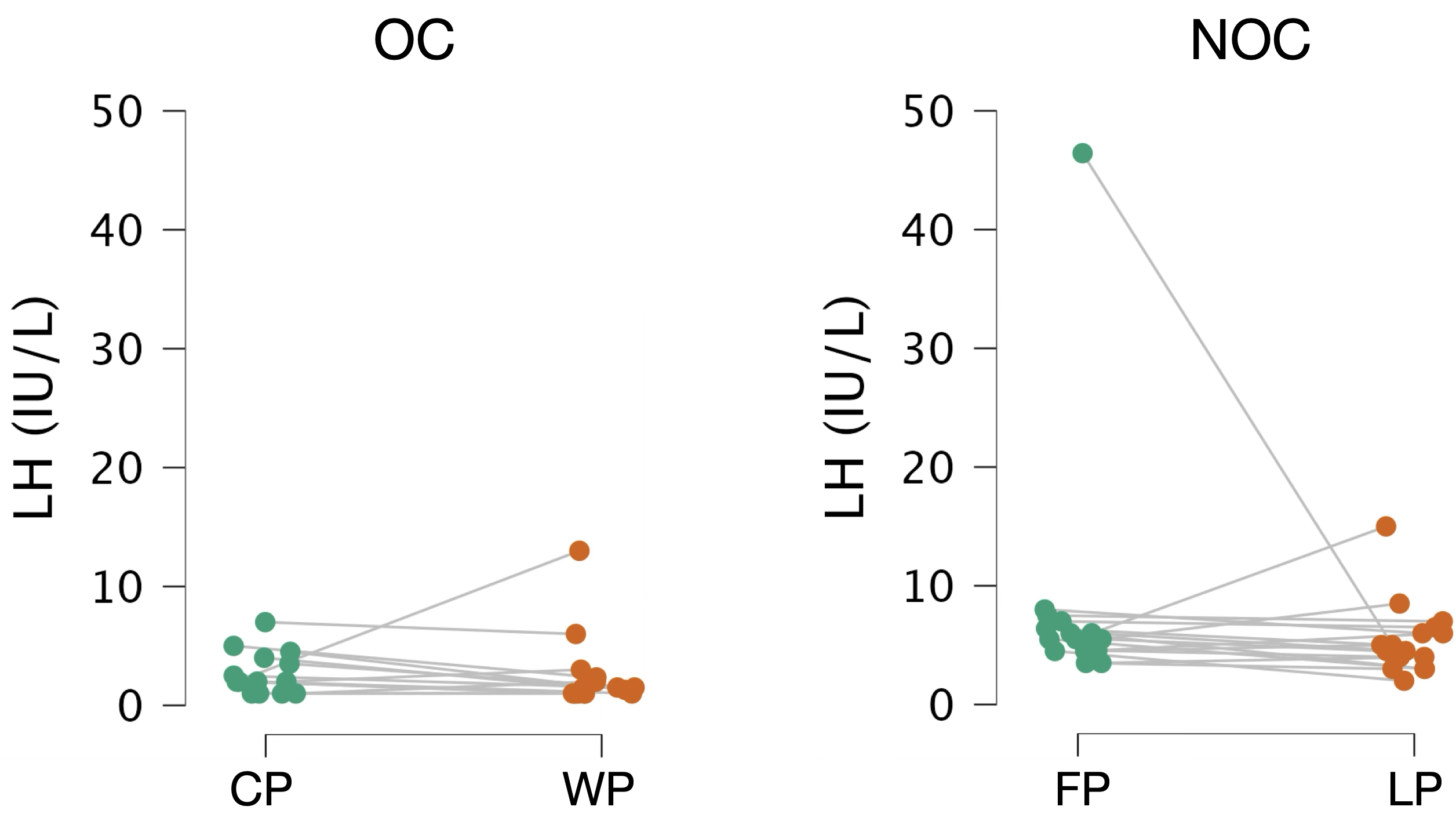


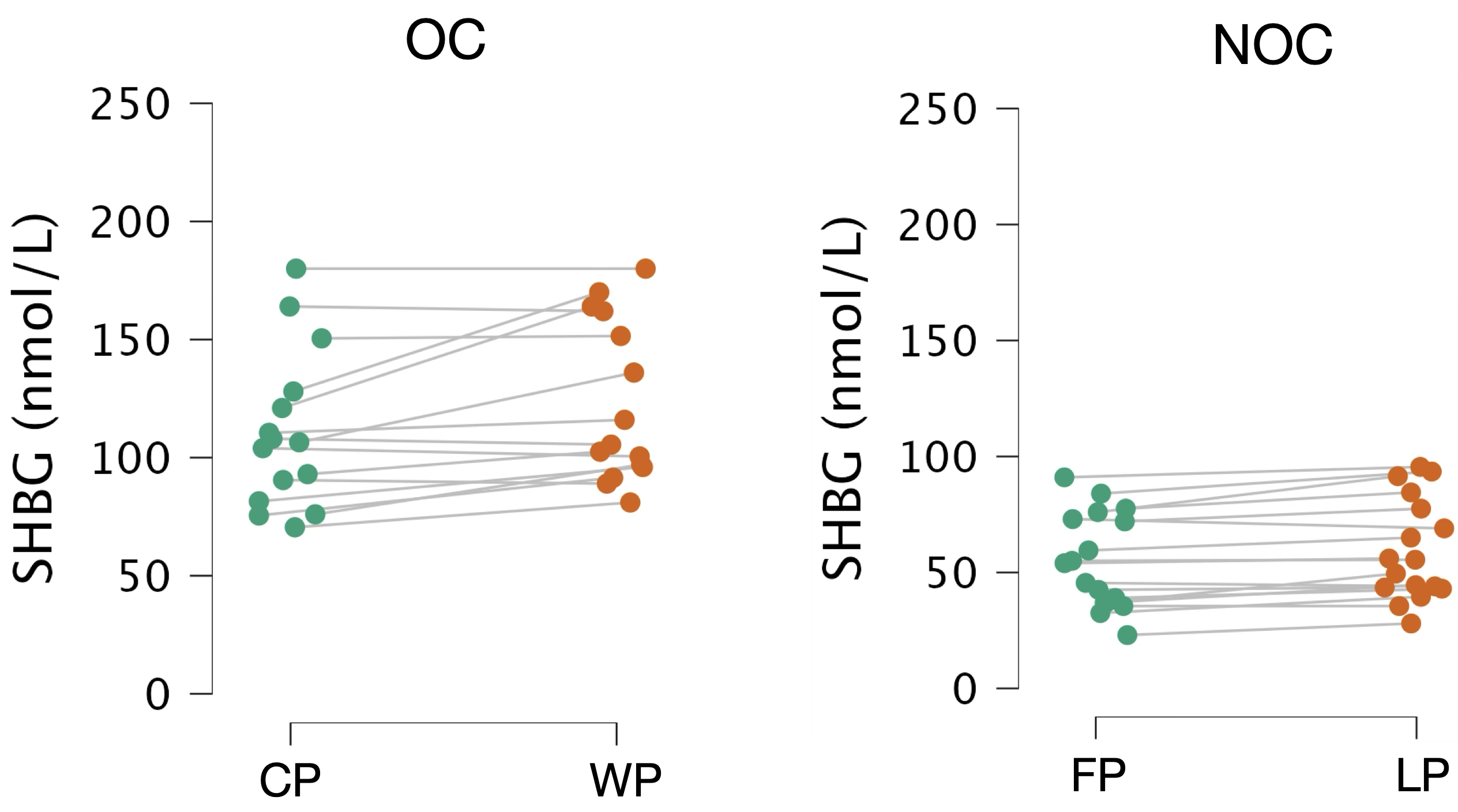


Fig. SI 3. Inter-individual variations in serum hormonal levels of follicle-stimulating hormone (FSH), luteinizing hormone (LH), and sex hormone binding globulin (SHBG). OC: oral contraceptives users, NOC: non-oral contraceptives users, CP: active pill consumption phase, WP: pill withdrawal phase, FP: follicular phase, LP: luteal phase.
